# HNF1**α** maintains pancreatic **α** and **β** cell functions in primary human islets

**DOI:** 10.1172/jci.insight.170884

**Published:** 2023-12-22

**Authors:** Mollie F. Qian, Romina J. Bevacqua, Vy M.N. Coykendall, Xiong Liu, Weichen Zhao, Charles A. Chang, Xueying Gu, Xiao-Qing Dai, Patrick E. MacDonald, Seung K. Kim

**Affiliations:** 1 Department of Developmental Biology, Stanford University School of Medicine, Stanford, California, USA.; 2 Diabetes, Obesity and Metabolism Institute, Icahn School of Medicine at Mount Sinai, New York, New York, USA.; 3 Department of Pharmacology and; 4 Alberta Diabetes Institute, University of Alberta, Edmonton, Canada.; 5 Stanford Diabetes Research Center,; 6 Departments of Medicine and Pediatrics (Endocrinology), and; 7 Northern California JDRF Center of Excellence, Stanford University School of Medicine, Stanford, California, USA.

**Keywords:** Endocrinology, Genetics, Beta cells, Diabetes, Monogenic diseases

## Abstract

*HNF1A* haploinsufficiency underlies the most common form of human monogenic diabetes (HNF1A–maturity onset diabetes of the young [HNF1A-MODY]), and hypomorphic *HNF1A* variants confer type 2 diabetes risk. But a lack of experimental systems for interrogating mature human islets has limited our understanding of how the transcription factor HNF1α regulates adult islet function. Here, we combined conditional genetic targeting in human islet cells, RNA-Seq, chromatin mapping with cleavage under targets and release using nuclease (CUT&RUN), and transplantation-based assays to determine HNF1α-regulated mechanisms in adult human pancreatic α and β cells. Short hairpin RNA–mediated (shRNA-mediated) suppression of *HNF1A* in primary human pseudoislets led to blunted insulin output and dysregulated glucagon secretion after transplantation in mice, recapitulating phenotypes observed in patients with diabetes. These deficits corresponded with altered expression of genes encoding factors critical for hormone secretion, including calcium channel subunits, ATPase transporters, and extracellular matrix constituents. Additionally, *HNF1A* loss led to upregulation of transcriptional repressors, providing evidence for a mechanism of transcriptional derepression through HNF1α. CUT&RUN mapping of HNF1α DNA binding sites in primary human islets imputed a subset of HNF1α-regulated genes as direct targets. These data elucidate mechanistic links between *HNF1A* loss and diabetic phenotypes in mature human α and β cells.

## Graphical abstract

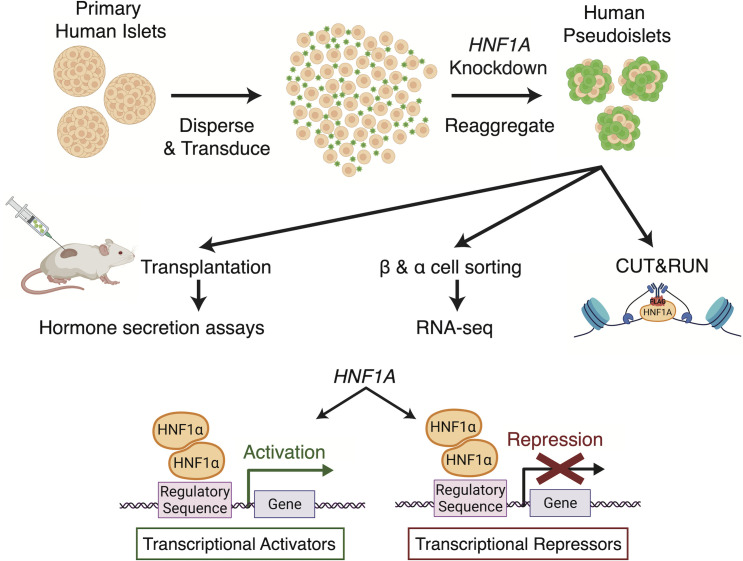

## Introduction

Diabetes mellitus is a pandemic disease of dysregulated glucose metabolism that arises from both acquired and genetic mechanisms. Understanding of diabetes genetics has been advanced by studies of monogenic diabetes, like maturity-onset diabetes of the young (MODY) (1), and identification of causal variants of type 2 diabetes (T2D) through genome-wide association studies (2). The most common MODY form, HNF1A-MODY, results from mutations in *HNF1A*, which encodes the transcription factor HNF1α (3). Moreover, hypomorphic *HNF1A* variants confer increased T2D risk (4). Despite the strong association between *HNF1A* deficiency and human diabetes, the mechanisms by which HNF1α regulates mature human islet cell function remain incompletely understood.

Previous studies of patients with HNF1A-MODY revealed impaired insulin secretion that improves with sulfonylurea treatment (5). These and studies in human stem cell models (6–10) strongly suggest a developmental role for HNF1α in islet β cell ontogeny and function, but the consequence of acute *HNF1A* loss in adult human islet β cells is unclear. *HNF1A* is also expressed in islet α cells (11, 12), and patients with HNF1A-MODY have dysregulated glucagon secretion (13, 14). However, the effect of *HNF1A* loss in adult human α cells is unknown. Thus, the roles of HNF1α in maintaining functions of mature α and β cells in human islets have not been firmly established.

This knowledge gap reflects challenges in studying HNF1α loss of function in adult islet cells. In humans, *HNF1A* haploinsufficiency underlies HNF1A-MODY, but mice heterozygous for an *Hnf1a*-null allele are not diabetic (15). Human studies using *HNF1A*-deficient β-like cells derived from multipotent stem cells have provided valuable insights regarding HNF1α regulation during islet cell development (6–10), but progeny cells from these studies did not fully recapitulate adult β cell functions, limiting conclusions about the roles of HNF1α in mature adult islets. Characterization of islets from a patient with HNF1A-MODY was recently reported (13), but conclusions in this case were inferred from studies of a single subject.

To elucidate how acute HNF1α loss leads to impaired function in mature human α and β cells, we used a pseudoislet-based strategy. Human pseudoislets are formed by dispersion and reaggregation of primary human islet cells, which permits efficient genetic targeting while maintaining cardinal features of primary islet cells (16). This approach enabled conditional genetic targeting, high-throughput RNA-Seq, cleavage under targets and release using nuclease (CUT&RUN) chromatin mapping, and transplantation-based functional assays of islet cells lacking HNF1α.

## Results

### shRNA targeting of HNF1A expression in primary human islets.

**Figure 1:**
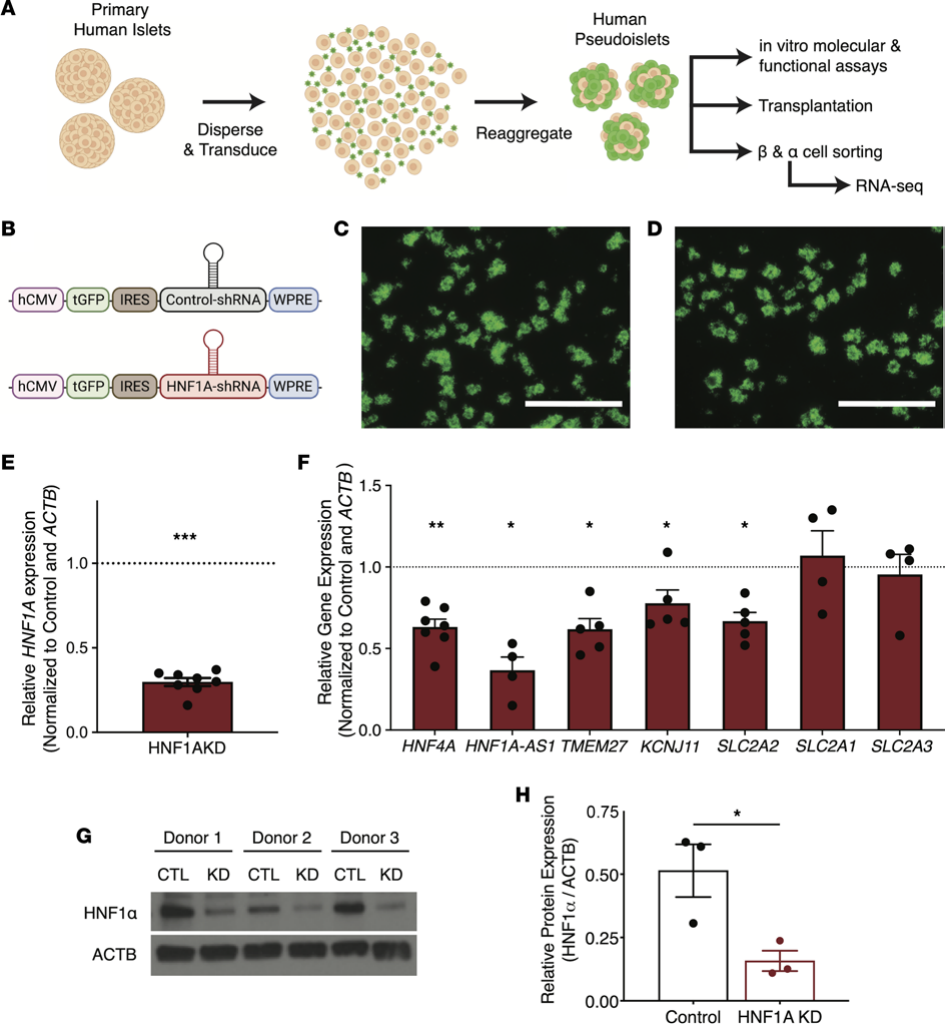
shRNA-mediated KD of *HNF1A* in primary human islets. (**A**) Formation of pseudoislets for downstream assays; transduction with lentivirus, followed by reaggregation over 5 days in culture. (**B**) Schematic of lentiviral constructs coding for shRNA and a GFP reporter (tGFP). Control-shRNA, nontargeting (“control”); HNF1A-shRNA, HNF1A-targeting (“HNF1AKD”). (**C** and **D**) Blue light (488 nm) images of human control (**C**) and HNF1AKD (**D**) pseudoislets. Scale bars: 1,000 μm. (**E** and **F**) mRNA expression of *HNF1A* (**E**) and putative HNF1α (**F**) targets in HNF1AKD relative to control pseudoislets; statistics performed on ΔCT values (*n* = 4–8 donors per gene). (**G**) Western blot analysis of HNF1α protein expression in control (CTL) and HNF1AKD (KD) pseudoislets (*n* = 3). (**H**) Quantification of blot intensities normalized to the housekeeping gene β-actin (ACTB). Data are presented as mean values ± SEM. Two-tailed *t* tests were used to generate *P* values; *P < 0.05, **P < 0.01, ***P < 0.00001.

We used the pseudoislet system (Figure 1A) to achieve conditional *HNF1A* loss in primary human islets. Specifically, primary human islets were dispersed to permit efficient transduction either with lentivirus expressing short hairpin RNA (shRNA) that suppressed *HNF1A* (HNF1A-knockdown [HNF1AKD]) or lentivirus expressing nontargeting control shRNA (control). Afterward, cells were reaggregated to form pseudoislets. The lentiviral vector coexpressed a *GFP* transgene (Figure 1B), thereby marking transduced cells. By 5 days after infection, we observed GFP^+^ cells (Figure 1, C and D) and a significant reduction in *HNF1A* mRNA as measured by quantitative PCR (qPCR) (Figure 1E). We also observed reduced mRNA levels encoding genes thought to be regulated by HNF1α, including *HNF4A*, *HNF1A-AS1*, *TMEM27*, *KCNJ11*, and *SLC2A2* (Figure 1F). While insulin mRNA levels were reduced after HNF1AKD, we did not detect significant reduction of glucagon mRNA or changes of insulin and glucagon protein expression (measured by ELISA) compared with controls (Supplemental Figure 1, A–D; supplemental material available online with this article; https://doi.org/10.1172/jci.insight.170884DS1). Western blot analysis verified significant reduction of HNF1α expression at the protein level in HNF1AKD versus control pseudoislets (Figure 1, G and H; see complete unedited blots in the supplemental material). Thus, lentiviral shRNA targeting with a pseudoislet strategy achieved conditional loss of *HNF1A* in primary human islet cells.

We next assessed hormone secretion after acute *HNF1A* loss. In static batch assays, insulin secretion from HNF1AKD pseudoislets was modestly but significantly reduced at 16.7 mM glucose 5 days after transduction (Supplemental Figure 1E). Likewise, glucagon secretion from HNF1AKD pseudoislets was blunted after stimulation with 1 mM glucose plus 10 mM l-arginine in vitro (Supplemental Figure 1F).

### HNF1α deficiency leads to phenotypes in transplanted α and β cells.

**Figure 2:**
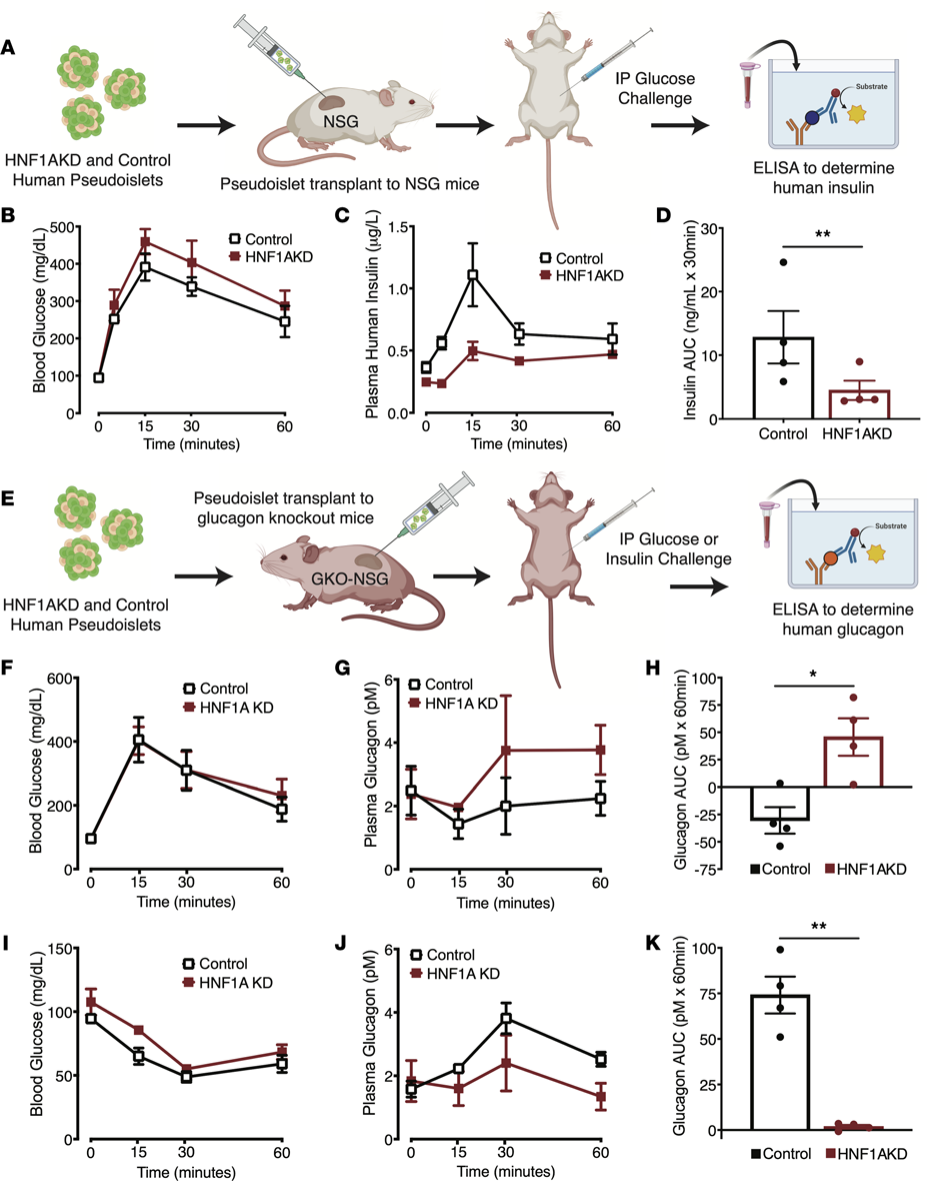
*HNF1A* suppression leads to dysregulated insulin and glucagon secretion after 1 month in vivo. (**A**) Experimental approach for control and HNF1AKD pseudoislet transplantation under kidney capsules of NSG mice and characterization of graft phenotypes; 1,000 pseudoislets were transplanted per mouse. (**B**–**D**) Blood glucose, plasma human insulin levels, and AUC of insulin excursion upon i.p. glucose challenge after transplantation of pseudoislets to NSG mice (*n* = 4 mice, 3 human donors). (**E**) Schematic of pseudoislet transplantation to glucagon-KO mice on an NSG background (GKO-NSG) for characterization of glucagon phenotypes; 1,000 pseudoislets were transplanted per mouse. (**F**–**K**) Blood glucose, plasma glucagon levels, and AUC of glucagon excursion upon i.p. glucose (**F**–**H**) or insulin (**I**–**K**) challenge after transplantation to GKO-NSG mice (*n* = 4 mice, 4 human donors). Data are mean values ± SEM. Two-tailed *t* tests were used to generate *P* values; *P < 0.05, **P < 0.01.

We predicted that hormone secretion phenotypes could be more pronounced after prolonged *HNF1A* suppression, but the duration of pseudoislet culture is limited to approximately 6 days. To extend the duration of *HNF1A* suppression and phenotyping in islet cells, we transplanted control and HNF1AKD pseudoislets under the renal capsules of immunocompromised NOD-scid IL2Rγnull (NSG) mice. Given previously reported species differences in *HNF1A*-deficiency phenotypes (15), the potential variability introduced by chemically induced diabetes, and the ability to distinguish between circulating human and mouse insulin via ELISA, we transplanted human pseudoislets in nondiabetic mice. The goal of these studies was to quantify human graft insulin secretion phenotypes after several weeks of *HNF1A* loss; we did not aim to characterize effects of grafts on host mouse metabolism. Four weeks after transplantation of human pseudoislets into nondiabetic NSG mice, we measured circulating human insulin (Figure 2A). After i.p. glucose tolerance testing (IP-GTT), we observed significant blunting of insulin secretion from transplanted HNF1AKD cells compared with controls (Figure 2, B–D). Moreover, this deficit was ameliorated by treatment of transplanted grafts with glibenclamide (Supplemental Figure 2, A–D), a sulfonylurea used in patients with diabetes with mutations in *HNF1A* (17). After recovery of human grafts, immunostaining demonstrated that HNF1α expression was significantly reduced in HNF1AKD pseudoislets (Supplemental Figure 2, E–G), confirming sustained *HNF1A* suppression after months in vivo. The total number of transduced (GFP^+^) cells recovered was similar between control and HNF1AKD grafts, suggesting that observed insulin secretion phenotypes were not due to differential graft survival (Supplemental Figure 2H). Thus, our transplantation studies revealed that conditional targeting and loss of *HNF1A* led to reduced human β cell insulin secretion.

Dysregulated glucagon secretion has also been documented in a subset of patients with HNF1A-MODY (13, 14). To measure glucagon secretion from transplanted human islets, we previously developed an NSG immuncompromised mouse harboring a glucagon gene KO (GKO-NSG) (18). Elimination of host glucagon production in GKO-NSG mice permits ELISA-based detection of glucagon from transplanted human α cells (Figure 2E). We transplanted HNF1AKD and control pseudoislets in GKO-NSG mice and then assessed glucagon secretion 4 weeks after transplantation. Glucose challenge (IP-GTT) resulted in comparable levels of acute hyperglycemia in GKO-NSG mice transplanted with HNF1AKD or control pseudoislets (Figure 2F). As expected, glucagon secretion from control grafts decreased in response to hyperglycemia by 30 minutes after glucose injection (Figure 2G). In contrast, glucagon secretion was not suppressed in HNF1AKD grafts, and AUC measures of net glucagon secretion were higher for HNF1AKD versus control grafts (Figure 2H). Thus, our transplantation studies also revealed inappropriate glucagon output by human α cells after acute *HNF1A* loss.

In addition to glucagon hypersecretion at high glucose levels, studies of islets from a single HNF1A-MODY donor reported reduced glucagon secretion in conditions that normally stimulate glucagon output (13). To investigate effects of *HNF1A* loss on glucagon secretion during hypoglycemia, we performed i.p. insulin tolerance tests (IP-ITT). IP-ITT led to acute hypoglycemia in GKO-NSG mice transplanted with HNF1AKD or control pseudoislets (Figure 2I). However, compared with controls, we observed blunted glucagon serum excursion in mice transplanted with HNF1AKD grafts (Figure 2, J and K). In summary, our functional studies reveal that acute loss of *HNF1A* in adult primary islets phenocopied multiple hormone secretion defects observed in humans chronically deficient for *HNF1A*, including reduced insulin secretion, excessive glucagon output during hyperglycemia, and blunted glucagon secretion during hypoglycemia.

### RNA-Seq identifies transcriptome changes after HNF1A loss in mature β cells.

**Figure 3:**
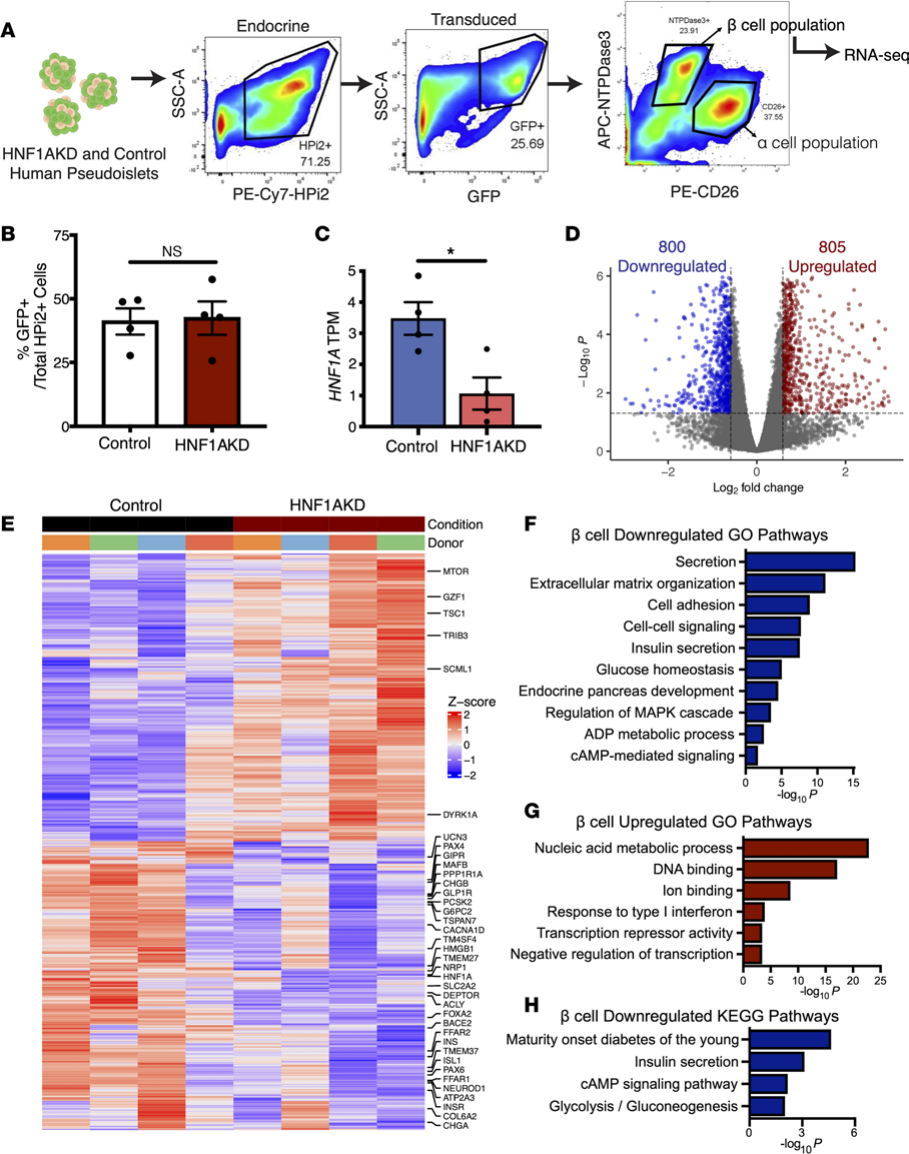
RNA-Seq of HNF1AKD β cells shows that HNF1α regulates insulin secretion, metabolism, developmental pathways, and cell-to-cell signaling in β cells. (**A**) Schematic of FACS scheme for isolation of transduced live β cells (HPi2^+^GFP^+^NTPDase3^+^) from control and HNF1AKD pseudoislets for downstream RNA-Seq (*n* = 4 donors). (**B**) Fraction of endocrine (HPi2^+^) cells expressing GFP in sorted samples. (**C**) *HNF1A* transcripts per million (TPM) in sequenced samples. (**D**) Differential expression analysis revealed significantly up- and downregulated genes after HNF1AKD in β cells. Fold change (FC) = 1.5, adjusted *P* = 0.05. (**E**) Heatmap of DEGs in β cells after HNF1AKD. (**F**–**H**) Significantly downregulated (**F**) and upregulated (**G**) Gene Ontology (GO) pathways and downregulated KEGG pathways (**H**) in HNF1AKD relative to control β cells. *P < 0.05.

HNF1α is a transcriptional regulator (3). Thus, to investigate the mechanisms underlying islet phenotypes we observed after *HNF1A* loss, we performed RNA-Seq to characterize the β cell transcriptome after *HNF1A* knockdown (Figure 3A). To isolate HNF1AKD β cells, we used FACS to purify lentivirus-infected GFP^+^ cells expressing NTPDase3, a human β cell surface marker (19). The fraction of endocrine cells (HPi2^+^) expressing GFP was similar between control and HNF1AKD samples (Figure 3B). Enrichment of insulin (*INS*) mRNA in NTPDase3^+^ fractions was confirmed by qPCR (Supplemental Figure 3, A and B). We produced and sequenced RNA-Seq libraries of GFP^+^NTPDase3^+^ cells from 4 human donors. Principal component analysis (PCA) showed clustering of samples by donor, consistent with prior studies (11, 20, 21), and separation of HNF1AKD and control samples (Supplemental Figure 3, C and D). The DESeq2 algorithm (22) identified 1,605 differentially expressed genes (DEGs) in β cells after *HNF1A* loss (Supplemental Table 3). This included 800 genes with significantly reduced mRNA, such as *HNF1A* (Figure 3C), and 805 with increased mRNA levels (Figure 3, D and E).

Gene Ontology (GO) pathway analysis of genes downregulated after *HNF1A* loss identified known regulators of hormone secretion (e.g., *CHGA*, *UCN3*), extracellular matrix (ECM) organization (*ADAMTS2*, *COL6A2*), cell-to-cell signaling (*GIPR*, *GLP1R*), glucose homeostasis (*G6PC2*, *SLC2A2*), and endocrine pancreas development (*MAFB*, *TM4SF4*) (Figure 3F). Additionally, downregulated genes were significantly enriched for KEGG pathways related to MODY (*HNF4A*, *INS*, *NEUROD1*, *GCK*) and cAMP signaling (*ADCY1*, *PDE3B*) (Figure 3H). By contrast, genes upregulated upon *HNF1A* loss were related to type I IFN responses (*IRF7*, *ISG20*) and transcription repressor activity (*GZF1*, *SCML1*) (Figure 3G). These results suggest that *HNF1A* is necessary in mature islet cells to maintain the expression of hundreds of crucial genes encoding hallmark regulators of human β cell function.

### RNA-Seq identifies adult human α cell transcriptome changes after HNF1A loss.

**Figure 4:**
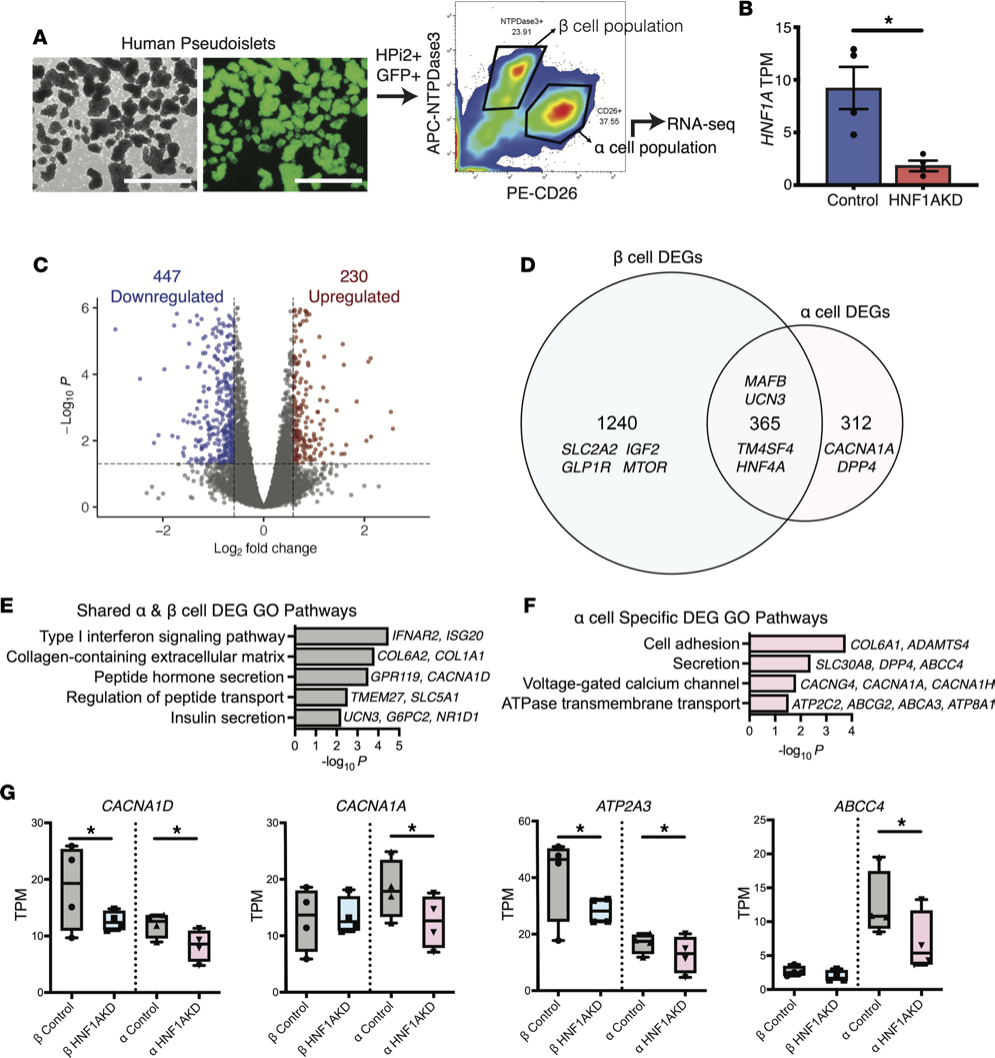
RNA-Seq of HNF1AKD α cells identifies dysregulation of calcium channel complexes and ATPase-coupled transmembrane transport as well as hormone secretion pathways shared with β cells. (**A**) Schematic of methods for isolation of transduced α cells (HPi2^+^GFP^+^CD26^+^) from control and HNF1AKD pseudoislets for downstream RNA-Seq (*n* = 4 donors); left image is bright-field, and right image is blue light (488 nm) of human HNF1AKD pseudoislets. Scale bars: 1,000 μm. (**B**) *HNF1A* transcripts per million (TPM) in sequenced α cell samples. (**C**) DEG analysis revealed significantly up- and downregulated genes after HNF1AKD in α cells. FC = 1.5, adjusted *P* = 0.05. (**D**) Venn diagram comparing α versus β cell DEGs revealed shared and cell-specific consequences of HNF1AKD. (**E** and **F**) Gene Ontology (GO) pathways of shared (**E**) and α cell (**F**) enriched DEG sets. (**G**) Box plots displaying TPM of select DEGs. *P < 0.05.

Our evidence of α cell dysregulation after HNF1AKD (Figure 2, F and K) and prior studies (13, 23) indicate that HNF1α is required for α cell function. However, little is known about HNF1α gene regulation in mature human α cells, aside from studies of a single patient-derived sample (13). Here we used RNA-Seq to investigate the *HNF1A*-dependent human α cell transcriptome. To isolate HNF1AKD α cells, we used FACS to enrich for transduced GFP^+^ cells expressing the human α cell marker CD26 (11, 24) (Figure 4A). Glucagon (*GCG*) enrichment in the CD26^+^ cell fraction was verified by qPCR (Supplemental Figure 3, A and B). Consistent with prior reports (11), analysis of RNA-Seq libraries generated from α cell fractions revealed that *HNF1A* mRNA was higher in control α versus β cells (Figure 3C and Figure 4B). Similar to β cells, we achieved greater than 50% suppression of *HNF1A* in α cells (Figure 4B).

DESeq2 analysis of HNF1AKD versus control RNA-Seq libraries demonstrated 447 downregulated DEGs and 230 upregulated DEGs after *HNF1A* loss in α cells (Figure 4C and Supplemental Table 4). More than half of α cell DEGs (365 of 677) were also dysregulated in β cells (Figure 4D). These included genes encoding previously characterized HNF1α targets (*TM4SF4*, *HNF4A*) and pancreatic islet regulators with species-specific expression patterns (*MAFB*, *UCN3*). GO term analysis of this overlapping gene set highlighted shared pathways related to type I IFN signaling (*IFNAR2*, *ISG20*), collagen-containing ECM (*COL6A2*, *COL1A1*), and peptide hormone secretion (*CACNA1D*, *SLC5A1*) (Figure 4E).

GO term analysis of DEGs also identified α cell–specific changes after *HNF1A* loss, including enrichment of pathways related to cell adhesion (*COL6A1*, *ADAMTS4*) and hormone secretion (e.g., the α cell–enriched factor *ABCC4*) (Figure 4F). Additional GO pathways enriched in α cell–specific DEGs included voltage-gated calcium (Ca^2+^) channel constituents and ATPase-coupled transmembrane transport (Figure 4F). While *CACNA1D* and *ATP2A3* expression were significantly downregulated in both β and α cells after *HNF1A* loss, expression of several additional voltage-gated Ca^2+^ channel (*CACNA1A*, *CACNG4*, *CACNA1H*) and ATPase-coupled transporter (*ABCC4*, *ABCG2*, *ABCA3*, *ATP8A1*) genes was significantly changed in α but not β cells (Figure 4G). Furthermore, patch-clamp electrophysiology studies revealed reduced Ca^2+^ currents in HFN1AKD versus control α cells, but not β cells, at 5 days after transduction (Supplemental Figure 4, A–C). Thus, our studies provide evidence for HNF1α direct or indirect regulation of hundreds of human pancreatic α cell genes, including genes not previously reported as HNF1α dependent, and a subset known to govern crucial physiologic processes that regulate hormone secretion.

### Direct targets of HNF1α identified by CUT&RUN.

**Figure 5:**
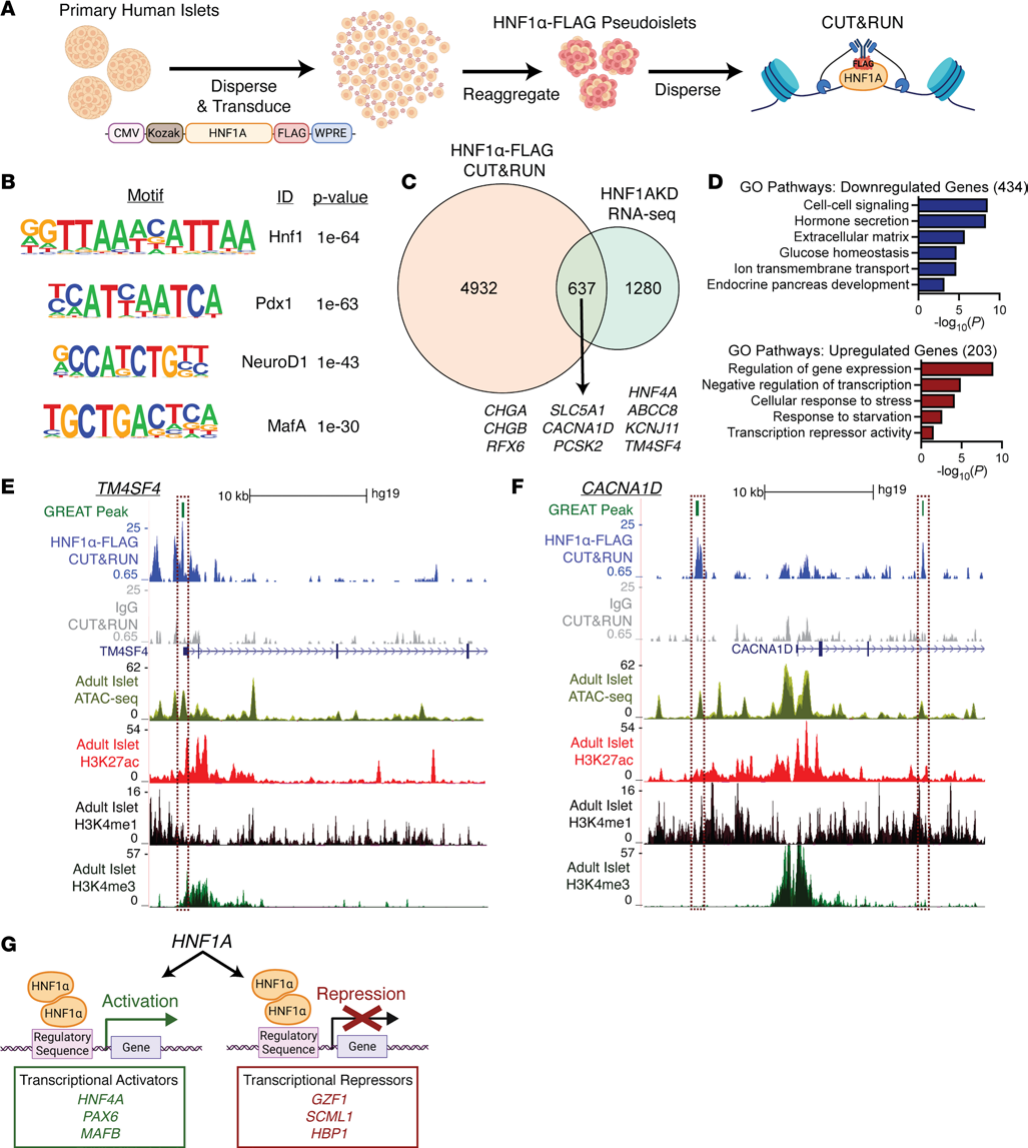
CUT&RUN identifies direct binding targets of HNF1α in primary human islet cells. (**A**) Schematic of methods. Pseudoislets expressing HNF1α-FLAG were used for CUT&RUN with anti-FLAG or anti-IgG (control) antibody (*n* = 3 donors). (**B**) Enriched motifs in the HNF1α-FLAG CUT&RUN peaks (versus IgG controls). (**C**) Venn diagram of genes associated with HNF1α-FLAG peaks identified by CUT&RUN (HNF1α-FLAG CUT&RUN) versus HNF1AKD DEGs in primary islet cells identified by RNA-Seq (HNF1AKD RNA-Seq). (**D**) Gene Ontology (GO) pathway analysis of overlapping genes from **C**, subset into genes that were downregulated or upregulated in RNA-Seq analysis. (**E** and **F**) UCSC Genome Browser tracks showing genomic regions associated with HNF1α-FLAG CUT&RUN peaks near the genes *TM4SF4* (**E**) and *CACNA1D* (**F**); HNF1α-FLAG CUT&RUN enriched peaks identified by Genomic Regions Enrichment of Annotations Tool (GREAT) are highlighted in dashed boxes, and regulated genes are depicted below IgG control tracks. Accessible chromatin regions in human islets are shown by ATAC-Seq and ChIP-Seq (H3K427ac, H3K4me1, and H3Kme3) tracks (27). (**G**) Schematic depicting HNF1α’s dual role as a transcriptional activator and repressor in pancreatic islet cells.

CUT&RUN assesses transcription factor DNA binding sites in situ (25), and we used this assay to identify direct HNF1α target genes in primary human islet cells. To overcome low endogenous islet expression of HNF1α and the reduced yields inherent to primary pancreatic samples, we used lentiviral transduction to express a transgene encoding human *HNF1A* tagged with the FLAG immunoepitope in human islet cells (Figure 5A). DNA bound by HNF1α-FLAG protein was enriched with an anti-FLAG antibody and sequenced. This approach captured HNF1α-FLAG–bound and immediately adjacent DNA regions. We used HOMER (26) to identify genomic regions captured by HNF1α-FLAG as previously reported (21). We observed enriched read densities in HNF1α-FLAG CUT&RUN DNA peak centers compared with minimal enrichment at these sites for IgG control samples (Supplemental Figure 5, A and B). HOMER analysis identified that HNF1α-FLAG–bound genomic peaks were significantly enriched for the HNF1α binding motif (Figure 5B) and other transcription factor motifs previously observed in pancreatic islet enhancer clusters (27), including PDX1 (Figure 5B) and NKX6.1 (Supplemental Figure 5C).

Using the Genomic Regions Enrichment of Annotations Tool (GREAT) algorithm (28), we associated HNF1α-FLAG–bound regions to 5,569 proximate genes. To prioritize direct regulatory targets of HNF1α, we compared these genes with DEGs identified by RNA-Seq after *HNF1A* loss. Of 1,917 DEGs in HNF1AKD α or β cells, 637 were also present in the HNF1α-FLAG CUT&RUN gene set (Figure 5C and Supplemental Table 5). The concordance between HNF1α-FLAG–bound regions and DEGs after *HNF1A* loss provides evidence that our CUT&RUN approach identified direct HNF1α targets.

Consistent with reports that HNF1α functions as a transcriptional activator (7, 29, 30), 68% (434/637) of the putative HNF1α targets identified by the intersection of our HNF1α-FLAG CUT&RUN and HNF1AKD RNA-Seq gene sets were downregulated upon *HNF1A* loss (Figure 5D). These downregulated genes were enriched for GO pathways related to cell-to-cell signaling (*CASR*, *DPP4*), hormone secretion (*ABCC8*, *KCNJ11*), ECM (*COL6A3*, *ADAMTS2*), glucose homeostasis (*G6PC2*, *GCK*), ion transmembrane transport (*ATP2A3*, *CACNA1D*), and endocrine pancreas development (*HNF4A*, *MAFB*). As expected, HNF1α-FLAG–bound genomic regions localized to presumptive accessible promoter and enhancer regions, as revealed by colocalization with transposase integration sites and histone marks reported in prior ATAC-Seq and ChIP-Seq studies (Figure 5, E and F) (27, 31). These findings support a model for direct HNF1α activation of genes, including those encoding factors essential for mature α and β cell function (Figure 5G). By contrast, the 203 (32%) putative HNF1α targets upregulated after *HNF1A* loss were broadly related to negative gene regulation (*NR1D1*, *GZF1*, *HBP1*, *SCML1*) (Figure 5D). These findings implicate HNF1α as a direct negative regulator of transcriptional repressors, suggesting that HNF1α derepression of transcriptional networks is another mechanism for maintaining human islet cell function (Figure 5G).

### Comparing islet transcriptomes after acute HNF1A loss and in congenital HNF1A-MODY.

**Figure 6:**
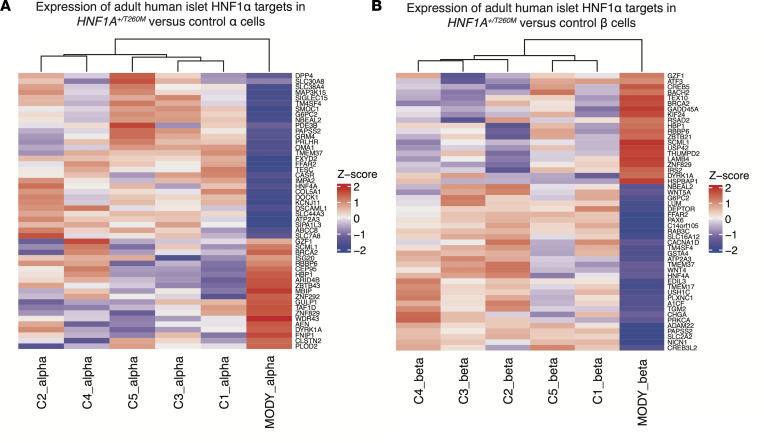
Comparison of HNF1α targets in primary human islets with HNF1A-MODY adult donor data sets demonstrates conserved HNF1α regulatory pathways that are critical for mature islet cell function. (**A** and **B**) Heatmaps showing relative expression of genes in α (**A**) and β (**B**) cells isolated from an *HNF1A^+/T260M^* donor (MODY) versus healthy control donors (C1-C5) (13); genes depicted were top DEGs in primary islet HNF1AKD RNA-Seq data also identified in HNF1α-FLAG CUT&RUN data (putative adult HNF1α targets).

To assess the applicability of studying acute *HNF1A* suppression in human pseudoislets in regard to understanding HNF1A-MODY, we compared HNF1α gene targets identified in this study with islet RNA-Seq data sets from a subject with HNF1A-MODY (13). The majority of HNF1α targets (368 of 637, 58%) we identified with CUT&RUN and RNA-Seq were differentially expressed in the RNA-Seq data obtained from human HNF1A-MODY islet cells. Pearson correlation analysis of normalized gene expression levels of these putative targets in our adult HNF1AKD α cells versus the HNF1A-MODY donor α cells revealed a significant positive correlation (*r* = 0.42, *P* = 2.2 × 10^–16^). Similarly, we observed a positive correlation in gene expression levels of putative HNF1α targets between HNF1AKD and HNF1A-MODY donor β cells (*r* = 0.32, *P* =5.3 × 10^–16^). This concordance between our data and those previously reported (13) is readily visualized in heatmaps of HNF1α target genes in α (Figure 6A) and β cells (Figure 6B). Notably, many of the genes common to our data set and the prior study were associated with ECM organization (*COL5A1*, *ADAM22*), ion transmembrane transport (*ATP2A3*, *SLC30A8*), glucose metabolism (*SLC2A2*, *G6PC2*), hormone secretion (*KCNJ11*, *CACNA1D*), and transcriptional repression (*SCML1*, *HBP1*, *BACH2*). Thus, our observations demonstrate some concordance of DEGs in α and β cells of islets from a congenital HNF1A-MODY donor and from acute conditional *HNF1A* loss of function in primary human islets.

## Discussion

Work here addresses knowledge gaps about the roles of HNF1α in mature pancreatic islet α and β cells. Prior studies of constitutive *HNF1A* deficiency have shaped our understanding of HNF1α roles in β-like cells derived from human stem cell lines (6–10). These cells retain features of fetal β cells; moreover, *HNF1A* targeting in these systems led to constitutive deficiency. Thus, while this prior work has broadened our understanding of HNF1α roles in islet β cell development, inferences about the roles of HNF1α in adult β cells are weakened by features of these stem cell models. Additionally, HNF1α function in mature α cells cannot be assessed in these systems. Here, we used conditional lentiviral shRNA targeting of *HNF1A* to investigate functions of HNF1α in primary human adult α and β cells, an approach for *HNF1A* study not previously reported. We observed dysregulated function and gene expression after acute *HNF1A* loss, and our transcriptome studies revealed that HNF1α regulates genes critical for establishing and maintaining characteristic islet cell features, like glucose metabolism and hormone secretion. These findings contribute to our understanding of adult islet cell gene regulation by HNF1α.

After suppression of *HNF1A*, we observed defects in insulin and glucagon secretion reminiscent of phenotypes in humans with *HNF1A* deficiency, including patients with HNF1A-MODY (13, 14) and those with T2D (4, 32). In contrast to recent growth in our understanding of mechanisms underlying *HNF1A-*deficient β cell dysfunction, little is known about the basis of phenotypes in α cells lacking *HNF1A* (13, 23). Work here revealed that α cell HNF1α is required to maintain expression of genes encoding known regulators of ECM organization, Ca^2+^ signaling, and ATPase-coupled transport. *HNF1A*-dependent expression of subsets of these genes was previously noted in human β cells or whole islets but not in purified α cells (6–10, 33). Prior publications have linked ECM signaling to regulation of insulin secretion (34, 35), and our work provides evidence that HNF1α promotes normal ECM dynamics in both β and α cells. Ca^2+^ influx is another well-established component of islet cell hormone secretion (36), and our studies support that HNF1α regulates voltage-gated Ca^2+^ channel subunit expression and Ca^2+^ channel currents in mature α cells. Store-operated Ca^2+^ flux has also been proposed to regulate glucagon secretion via intracellular sequestration of Ca^2+^ through sarco(endo)plasmic reticulum ATPases (SERCA) (37, 38). We found that several genes related to ATPase-coupled transport were downregulated after HNF1AKD in α cells, including *ATP2A3* and *ATP2C2*, which encode ATP-driven Ca^2+^ transporters (39, 40). These findings suggest that dysregulation of ATPase function and Ca^2+^ transport may contribute to anomalous glucagon secretion from α cells in *HNF1A*-deficient diabetes.

To identify direct genetic targets of HNF1α in human islet cells, we used CUT&RUN after misexpression of FLAG-tagged HNF1α. We recognize that results after misexpression of tagged HNF1α should be interpreted cautiously. Therefore, we also integrated CUT&RUN with DEG analysis after *HNF1A* suppression, and this combined approach increased confidence in “calling” direct genetic targets of HNF1α imputed by CUT&RUN. Thus, we identified hundreds of putative direct genetic targets of HNF1α that are critical to mature α and β cell functions.

Our analysis also provides index evidence that HNF1α may both activate and derepress gene networks in human islets (Figure 5G). While HNF1α is well known as a transcriptional activator (7, 29, 30), gene expression studies here and in prior reports demonstrate that HNF1α deficiency results in both decreased and increased gene expression (6, 7, 13, 30). Direct transcriptional repression by HNF1α has been observed in hepatocytes (41), but it has not previously been reported in islets. Here, we report that approximately one-third of HNF1α target genes were upregulated after *HNF1A* loss. Notably, many of these upregulated targets encode transcriptional repressors with previously characterized roles in repressing cell proliferation (HBP1) (42), homeotic gene expression (SCML1) (43), and antioxidant response pathways (BACH2) (44). Dynamic transcriptional derepression is critical for multiple physiological processes, including endocrine cell differentiation (45). Our findings support the view that islet phenotypes from *HNF1A* deficiency could reflect loss of transcriptional activation and repression in HNF1α-dependent genetic pathways.

In summary, our study identifies genetic targets of HNF1α regulation in primary human islets and correlates loss of *HNF1A* with dysregulated gene expression and functional deficits in α and β cells. A subset of these features phenocopy those in humans with diabetes from *HNF1A* deficiency. We demonstrate that HNF1α maintains genetic pathways crucial for regulated hormone secretion and derepresses pathways that may be necessary for mature islet function. These findings advance our understanding of *HNF1A*-dependent mechanisms that maintain adult human α and β cell function.

## Methods

Supplemental Methods are available online with this article.

### Human islet procurement.

Deidentified, nondiabetic human islets were obtained through the Integrated Islet Distribution Network, International Institute for the Advancement of Medicine, UCSF, and Alberta Diabetes Institute IsletCore (https://www.bcell.org/adi-isletcore.html). Supplemental Table 1 contains donor details.

### Constructs and lentivirus production.

Lentiviral constructs for shRNA targeting exon 4 of *HNF1A* were obtained from Dharmacon. pLenti-CMV-HNF1A-cMyc-DDK was used in CUT&RUN experiments (OriGene RC211201L1). Lentiviruses were produced by transfection of HEK293T cells with lentiviral constructs and pMD2.G and psPAX2 packaging constructs (Addgene). TurboFect reagents were used for transfection (Thermo Fisher Scientific), and supernatants were purified using PEG-it (System Biosciences).

### Human pseudoislet generation.

Human pseudoislets were generated as previously described (16, 20, 21). Briefly, intact human islets were dispersed into single cells by enzymatic digestion (Accumax, Invitrogen) and transduced with 1 × 10^9^ viral units/mL lentivirus. Transduced cells were cultured in ultra–low attachment cell culture plates (Corning) for 5 days prior to analysis.

### qPCR.

RNA was isolated from whole pseudoislets using the PicoPure RNA isolation kit (Invitrogen). cDNA was synthesized using the Maxima first strand kit (Thermo Fisher Scientific), and gene expression was assessed by PCR using TaqMan gene expression mix (Thermo Fisher Scientific) and probes listed in Supplemental Table 2.

### Western blot analysis.

Whole-cell protein extracts were obtained from 500 pseudoislets per sample through lysis in RIPA buffer (Thermo Fisher Scientific) containing 1× protease inhibitor cocktail (Roche). Protein concentrations were quantified using a NanoDrop spectrophotometer; 40 μg of total protein was mixed with sample buffer (4× Laemmli Buffer [Bio-Rad], 10% β-mercaptoethanol [Sigma-Aldrich]) and boiled for 5 min at 95°C. Samples were run on a 4%–15% Mini-PROTEAN TGX Precast Gel (Bio-Rad) for 60 minutes at 100 V in Tris-glycine–SDS buffer. Samples were then transferred to a polyvinylidene difluoride (PVDF) membrane at 180 mA for 40 minutes in Tris-glycine–methanol buffer. The PVDF membrane was blocked in 5% milk in phosphate-buffered saline containing 0.1% Tween-20 for 1 hour at room temperature. Incubation with a primary antibody against HNF1α (Rb anti-HNF1A, Abcam ab204306; 1:150) was performed at 4°C overnight followed by incubation with an HRP-conjugated anti-rabbit secondary antibody (HRP Dk anti-Rb, Thermo Fisher Scientific A16035; 1:750) for 1 hour at room temperature (see Supplemental Table 2 for antibody details). HRP signal was detected on x-ray films by chemiluminescent substrate (Thermo Fisher Scientific, 34577). Blots were stripped using Restore Western Blot Stripping Buffer (Thermo Fisher Scientific) and reprobed using an HRP-conjugated antibody against β-actin (HRP Ms anti–β-actin, Abcam ab49900 1:50,000). ImageJ (NIH) was used to quantify protein bands; HNF1α band intensity was normalized to β-actin (housekeeping gene) band intensity for each sample.

### Transplantation and in vivo assessment of pseudoislet function.

Batches of 1,000 pseudoislets were transplanted under the renal capsule of 3-month-old male NSG (The Jackson Laboratory, stock no. 005557) or GKO-NSG (18) mice using a microcapillary tube, as described previously (18). Four weeks later, mice received an i.p. injection of 3 g glucose/kg body weight. For sulfonylurea sensitivity testing, NSG mice received 2.5 mg glibenclamide/kg body weight (Sigma-Aldrich, G0639) via a single i.p. injection 6 weeks after transplantation of pseudoislets. For ITT, GKO-NSG mice received 0.5U Humulin R/kg body weight (Lilly) via i.p. injection. Blood samples were collected via the tail vein; glucose and hormones were measured using a glucometer (True Metrix) and ELISA kits (Mercodia).

### Patch-clamp electrophysiology studies.

Single-cell patch-clamp studies were performed as described previously (46). Pseudoislets were dissociated to single cells and cultured in 5.5 mM glucose media for 1–3 days. Prior to whole-cell patch clamping, media were changed to a bath solution containing 118 mM NaCl, 20 mM tetraethylammonium-Cl, 5.6 mM KCl, 1.2 mM MgCl_2_, 2.6 mM CaCl_2_, 5 mM HEPES, and 5 mM glucose (pH 7.4 with NaOH) (all chemical reagents from Sigma-Aldrich) in a heated chamber (Warner Instruments, TC-324B) (32°C–35°C). Patch clamping was performed using fire-polished thin-wall borosilicate pipettes coated with Sylgard (World Precision Instruments) (3–5 MOhm) containing 125 mM Cs-glutamate (Sigma-Aldrich), 10 mM CsCl (Sigma-Aldrich), 10 mM NaCl (Sigma-Aldrich), 1 mM MgCl_2_ (Sigma-Aldrich), 0.05 mM EGTA (Thermo Fisher Scientific), 5 mM HEPES (Sigma-Aldrich), 0.1 mM cAMP (Sigma-Aldrich), and 3 mM MgATP (Sigma-Aldrich) (pH 7.15 with CsOH from Sigma-Aldrich). Data were recorded using a HEKA EPC10 amplifier and PatchMaster Software (HEKA Instruments Inc.) within 5 minutes of break-in. The stability of seal (>10 GOhm) and access resistance (<15 MOhm) throughout the experiment was assessed for quality control. FitMaster (HEKA Instruments Inc) was used for data analysis.

### Extracellular staining and FACS of human islet cells.

Pseudoislets were dispersed into single cells, stained with the LIVE/DEAD Fixable Near-IR kit (Invitrogen), and washed with cell staining buffer (BioLegend). The following primary-secondary conjugated antibodies were used: HPi2-PE/Cy7 (Novus Biologicals NBP1-18946PECY7), NTPDase3-647 (Jean Sévigny’s lab clone hN3-B3_S_), and CD26-PE (BioLegend 302706) (Supplemental Table 2). Labeled cells were sorted on a special order 5-laser FACS Aria II (BD Biosciences) using a 100 μm nozzle, with compensation controls and doublet removal. Sorted cells were collected in 100 μL of FACS buffer with RiboLock RNase inhibitor (Thermo Fisher Scientific).

### RNA-Seq library preparation and data analysis.

Approximately 5,000 sorted live β or α cells were used for each RNA-Seq library construction. RNA was isolated using the PicoPure RNA isolation kit (Invitrogen). The SMART-Seq v4 Ultra Low input RNA kit (Clontech) was used to amplify cDNA, and libraries were generated using the Nextera XT DNA Library Preparation Kit (Illumina). Barcoded libraries were sequenced as paired-end 150 base pair (PE150) reads on the Illumina NovaSeq 6000 platform. All libraries had > 30 million reads, and FastQC v0.11.9 was used for quality control. Barcodes were trimmed using Trim Galore v0.5.0. Reads were aligned to the human genome index (GRCh38, Ensembl release 104) using STAR v2.6.1d (47). Estimated counts and transcripts per million (TPM) were quantified using RSEM v1.3.1 (48). DEGs were detected using the DESeq2 R package (22), controlling for donor differences; *P* adjusted cutoff of 0.05 and fold change threshold of 1.5 were used. g:Profiler version e106_eg53_p16_65fcd97 was used for gene set enrichment analysis (49), using the Benjamini-Hochberg false discovery rate method. RNA-Seq data sets from HNF1A-MODY donor islets were obtained from Haliyur et al. (13). Pearson’s correlation analysis was performed on Z-transformed average HNF1AKD TPMs (versus 4 controls) and Z-transformed values of the HNF1A-MODY donor (*n* = 1 HNF1A-MODY donor; *n* = 5 controls) for each cell type.

### CUT&RUN assay and library preparation.

CUT&RUN was performed on 500,000 dispersed HNF1α-FLAG pseudoislet cells per condition using CUTANA ChIC/CUT&RUN protocol v3.1. Nuclei were extracted with nuclear extraction buffer (20 mM HEPES-KOH [pH 7.9]; 10 mM KCl; 0.1% Triton X-100; 20% glycerol; 1 mM MnCl_2_; 0.5 mM spermidine; 1× Halt protease inhibitor; Thermo Fisher Scientific) for 10 minutes on ice and immobilized onto Concanavalin-A beads (EpiCypher). After blocking and washes, samples were incubated with 0.5 μg of rabbit anti-FLAG (MilliporeSigma F7425) or rabbit anti-IgG (EpiCypher 13-0042) antibodies (Supplemental Table 2) overnight at 4°C. pAG-MNase (EpiCypher) was added to nuclei (1:20) and incubated at room temperature for 10 minutes. Targeted chromatin digestion was induced by adding 100 mM CaCl_2_ and nutating for 2 hours at 4°C. DNA fragments were purified using the CUTANA ChIC/CUT&RUN kit, according to the manufacturer’s instructions. DNA was resuspended in 0.1× Tris-EDTA buffer solution and used for library preparation with the CUTANA CUT&RUN Library Prep Kit (EpiCypher, 14-1001), according to the v1 manual. Libraries were sequenced as PE150 reads on the NovaSeq platform.

### CUT&RUN data analysis.

All libraries had > 25 million reads. Reads were trimmed and aligned using CUT&RUNtools (50). Trimmomatic was used for trimming (51), Bowtie2 for alignment (52), and HOMER for peak calling using macs2.narrow outputs (26). *P* values for motif enrichment were generated by HOMER software. Genome browser tracks were generated from mapped reads using the “makeUCSCfile” command. The GREAT algorithm was used for gene annotation, using default parameters (28).

### Statistics.

The number of biological replicates, measure of central tendency/deviation, and statistical test used for analysis are detailed in figure legends. Specifically, 2-tailed *t* tests were used to generate *P* values for comparison of ΔCT values in qPCR data. Two-tailed t tests were also used to generate *P* values for comparison of AUC of hormone excursion from pseudoislets after transplantation to appropriate mouse models. Additional details regarding RNA-Seq and CUT&RUN data analyses are included in respective Methods subsections above. Graphs and statistical analyses were produced using GraphPad Prism (v9) and R (v4.1.1). Cytometry data were graphed using FlowJo (v10.8). Venn diagrams and heatmaps were generated in R. Browser tracks were generated using the UCSC genome browser (53), and method graphics were created with BioRender.com.

### Study approval.

Animal studies were approved by Stanford’s Administrative Panel on Laboratory Animal Care (APLAC, 29985). Human pancreatic samples were deidentified and, therefore, not considered as human subject research by the Stanford IRB.

### Data availability.

The RNA-Seq and CUT&RUN sequencing data from this publication have been deposited in NCBI’s Gene Expression Omnibus (GEO) (54) and are accessible through GEO Series accession no. GSE246230. Values for all data points shown in graphs are in the Supporting Data Values file.

## Author contributions

MFQ and SKK conceptualized the study. MFQ, RJB, VMNC, XL, WZ, CAC, XG, and XQD performed experiments. MFQ and SKK wrote the manuscript with input from all coauthors. SKK supervised the study. MFQ, RJB, VMNC, PEM, and SKK acquired the funding.

## Supplementary Material

Supplemental data

Supplemental tables 3-5

Supporting data values

## Version 1 11/09/2023

In-Press Preview

## Version 2 12/22/2023

Electronic publication
