# Supplementary material for: HNF1α maintains pancreatic α and β cell functions in primary human islets
Source: JCI Insight. 2023 Dec 22;8(24):e170884. doi: 10.1172/jci.insight.170884 (PMC10807710; doi:10.1172/jci.insight.170884)
Supplement: Supplemental data [file jciinsight-8-170884-s091.pdf]

SUPPLEMENTAL MATERIAL

HNF1α maintains pancreatic α and β cell functions in primary human islets

Mollie F. Qian, Romina J. Bevacqua, Vy M.N. Coykendall, Xiong Liu, Weichen Zhao, Charles A. Chang, Xueying Gu, Xiao-Qing Dai, Patrick E. MacDonald, Seung K. Kim

SUPPLEMENTAL METHODS

In vitro Hormone Secretion Assays

Batches of 30 pseudoislets were used for in vitro secretion assays as previously described (Peiris et al. 2018). Pseudoislets were incubated at 2.8mM then 16.7mM glucose or 6mM glucose then 1mM glucose+10mM L-arginine for 60min, and supernatants collected. Supernatant and lysate hormones were quantified using ELISA kits (Mercodia).

Immunofluorescence staining

Mouse kidneys containing recovered human pseudoislet grafts were fixed in 4% paraformaldehyde for 1.5hrs at 4°C. Samples were then dehydrated with 30% sucrose and embedded in OCT. Six-µm thick frozen sections were mounted onto slides and stained using standard cryostaining procedures. For immunostaining, sections were washed in PBS, permeabilized for 10 minutes using 0.2% Triton X, and incubated with blocking solution (5% normal donkey serum and 0.1% Triton X in PBS) for 1hr before overnight incubation with primary antibodies in blocking buffer at 4°C. The following primary antibodies were used: guinea pig anti-Insulin (1:200; Dako A0564) and rabbit anti-HNF1A (1:1000, Abcam ab204306). Slides were washed with PBS, incubated with Alexa Flour-conjugated secondary antibodies (555 or 647; 1:500, donkey-anti-primary-host, Jackson ImmunoResearch) at room temperature for 1hr, incubated with Hoechst 33342 (Thermo Fisher Scientific, 1:2000) for 5min to detect nuclei, and preserved with mounting medium (Vector Labs, Vectashield H-1200). Images were obtained using a Zeiss AxioM1 microscope and Leica SP2 confocal microscope.

The ImageJ plug-in EzColocolization (Stauffer et al. 2018) was used for quantification of $GFP^{+}HNF1A^{+}$ and $GFP^{+}DAPI^{+}$ cells from grafts.

References

Peiris H, et al. Discovering human diabetes-risk gene function with genetics and physiological assays. *Nat Commun*. 2018;9(1):3855.
Stauffer W, Sheng H, Lim HN. EzColocalization: An ImageJ plugin for visualizing and measuring colocalization in cells and organisms. *Sci Rep* 2018;8: 15764.

SUPPLEMENTAL TABLES AND FIGURES

Table S1: Human Islet Donor Information

**Source**	**Sample ID**	**Age**	**Sex**	**BMI**	**HbA1c**
IIDP	SAMN20478103	30	Male	25.4	5.8%
UCSF	rHIP148	38	Female	32.6	5.4%
ADI	R412	42	Male	29.9	4.8%
IIAM	SIRCPilot4	15	Female	22.9	5.3%
ADI	R440	35	Female	26.7	3.8%
UCSF	rHIP152	32	Female	29.2	5.0%
IIDP	SAMN28673685	37	Male	28.4	5.3%
IIAM	SIRC-CC	18	Female	35	Nondiabetic
IIAM	SIRC-1	44	Female	23.8	Nondiabetic
IIDP	SAMN18092805	56	Male	21.6	5.1%
IIAM	SIRC-3	16	Male	35	5.5%
UCSF	rHIP150	36	Female	27.9	5.6%
IIDP	SAMN28673685	37	Male	28.4	5.3%
IIDP	SAMN28867622	36	Male	29.6	5.4%
ADI	R352	64	Female	20.4	5.3%
ADI	R357	64	Male	24.3	5.1%
IIDP	SAMN14132340	31	Male	27	5.2%
UCSF	rHIP141	41	Male	25.2	5.5%
IIDP	SAMN15770453	48	Female	30.9	5.8%
ADI	R421	60	Female	25.9	4.8%
UCSF	rHIP144	47	Female	23.5	5.7%
ADI	R373	31	Male	27.5	4.4%
ADI	R450	66	Female	21.9	4.3%
ADI	R447	62	Male	30.2	5.7%

Abbreviations: BMI= Body Mass Index (in kg/m2), HbA1c= Hemoglobin A1C, IIDP= Integrated Islet Distribution Network, UCSF= University of California San Francisco, ADI= Alberta Diabetes Institute IsletCore, IIAM= International Institute for the Advancement of Medicine. Age is represented in years.

Table S2: Reagents List

**Reagent Type**	**ID**	**Source**	**Product Number**
RT-qPCR Taqman Probe	*ACTIN-B*	Thermo Fisher Scientific	Hs4352667_m1
RT-qPCR Taqman Probe	*GCG*	Thermo Fisher Scientific	Hs01031536_m1
RT-qPCR Taqman Probe	*HNF1A*	Thermo Fisher Scientific	Hs01551750_m1
RT-qPCR Taqman Probe	*HNF1A-AS1*	Thermo Fisher Scientific	Hs00703760_s1
RT-qPCR Taqman Probe	*HNF4A*	Thermo Fisher Scientific	Hs00604435_m1
RT-qPCR Taqman Probe	*INS*	Thermo Fisher Scientific	Hs00355773_m1
RT-qPCR Taqman Probe	*KCNJ11*	Thermo Fisher Scientific	Hs00265026_s1
RT-qPCR Taqman Probe	*SLC2A1*	Thermo Fisher Scientific	Hs00892681_m1
RT-qPCR Taqman Probe	*SLC2A2*	Thermo Fisher Scientific	Hs01096904_m1
RT-qPCR Taqman Probe	*SLC2A3*	Thermo Fisher Scientific	Hs00359840_m1
RT-qPCR Taqman Probe	*TMEM27*	Thermo Fisher Scientific	Hs00252907_m1
Western Blot Antibody	Rb anti-HNF1A	Abcam	ab204306
Western Blot Antibody	HRP Dk anti-Rb	Thermo Fisher Scientific	A16035
Western Blot Antibody	HRP Ms anti-β-actin	Abcam	ab49900
FACS Antibody	HPi2-PE/Cy7	Novus Biologicals	NBP1-18946PECY7
FACS Antibody	NTPDase3-647	Jean Sévigny Lab	clone hN3-B3S
FACS Antibody	CD26-PE	BioLegend	302706
CUT&RUN Antibody	Rb anti-FLAG	Sigma-Millipore	F7425
CUT&RUN Antibody	Rb anti-IgG	EpiCypher	13-0042

Table S3: β cell RNA-seq Differentially Expressed Genes (*P*adj<0.05, FC>1.5 or <-1.5) (see .xlsx)

Table S4: α cell RNA-seq Differentially Expressed Genes (*P*adj<0.05, FC>1.5 or <-1.5) (see .xlsx)

Table S5: Direct regulatory targets of HNF1α in human islet cells (overlap of genes identified by CUT&RUN and HNF1AKD differentially expressed genes) (see .xlsx)

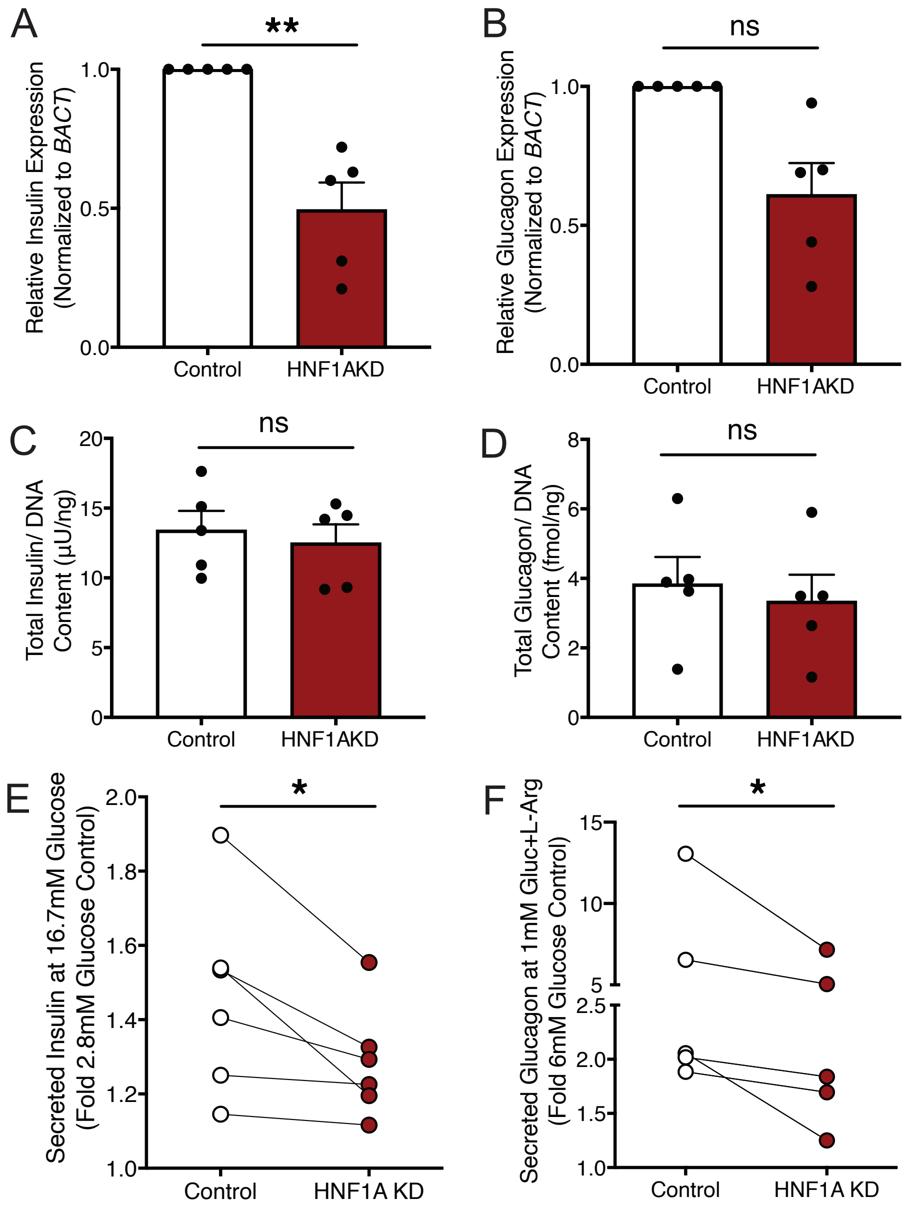

Figure S1. Assessment of pseudoislet hormone content and in vitro secretion. (A) Insulin (n=5) and (B) glucagon (n=5) mRNA expression by RT-qPCR; statistics performed on deltaCT values. Total (C) insulin protein content (n=5) and (D) glucagon protein content (n=5) in primary human pseudoislets by ELISA 5 days after transduction with lenti-Control-shRNA ("Control") or lenti-HNF1A-shRNA ("HNF1AKD"). Secreted (E) insulin (n=6) and (F) glucagon (n=5) from pseudoislet static batch assays by ELISA 5 days after transduction; lines connect samples from the same donor; Gluc=Glucose and L-Arg=10mM L-Arginine. Error bars represent SEM. Two-tailed t-tests paired by donor were used to generate p-values; **P<0.01, *P<0.05, ns= not statistically significant.

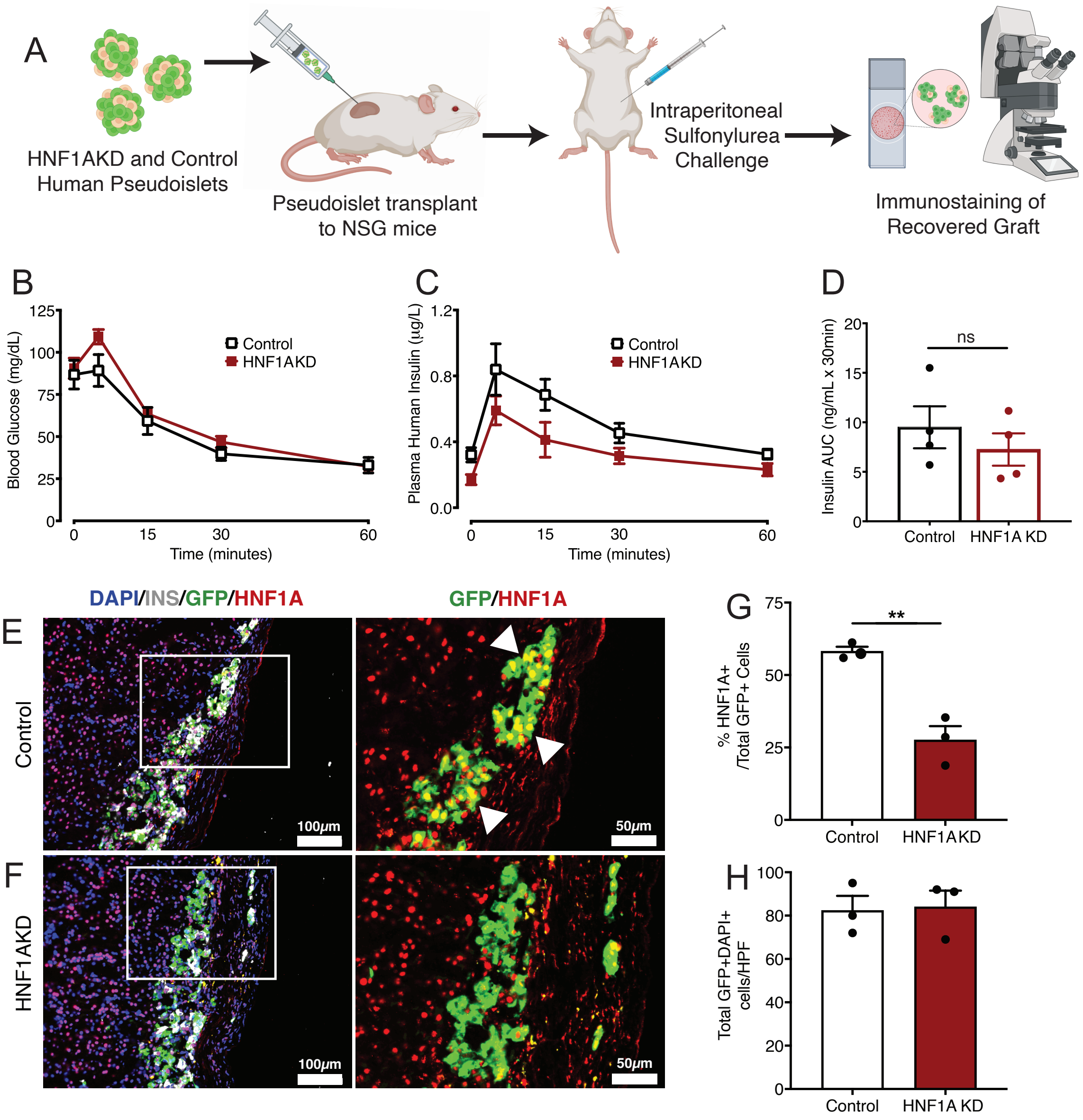

Figure S2. HNF1AKD is durable after transplantation and insulin phenotypes can be ameliorated with sulfonylurea treatment in vivo. (A) Schematic of experimental approach for pseudoislet transplantation under murine kidney capsules and characterization of in vivo phenotypes; “HNF1AKD” = pseudoislets transduced with lenti-HNF1A-shRNA; “Control” = pseudoislets transduced with lenti-Control-shRNA. (B) Blood glucose, (C) plasma human insulin levels, and (D) area under the curve (AUC) of insulin excursion (not significant; *P*=0.44) upon intraperitoneal sulfonylurea challenge (n=4 mice, 3 human islet donors per condition). (E-G) Immunostaining of recovered grafts from mice transplanted with (E) Control or (F) HNF1AKD pseudoislets; right-hand panels are magnification of boxed regions in left-hand panels; arrow heads point to yellow areas of overlap indicating $GFP^{+}HNF1A^{+}$ cells. Background mouse host kidney cells express HNF1A but lack GFP. (G) Quantification of $GFP^{+}HNF1A^{+}$ cells from grafts. (H) Total number of $GFP^{+}DAPI^{+}$ cells per high-power field (HPF) from grafts. Data are presented as mean values ± SEM. Two-tailed t-tests were used to generate *P*-values; ***P*<0.01.

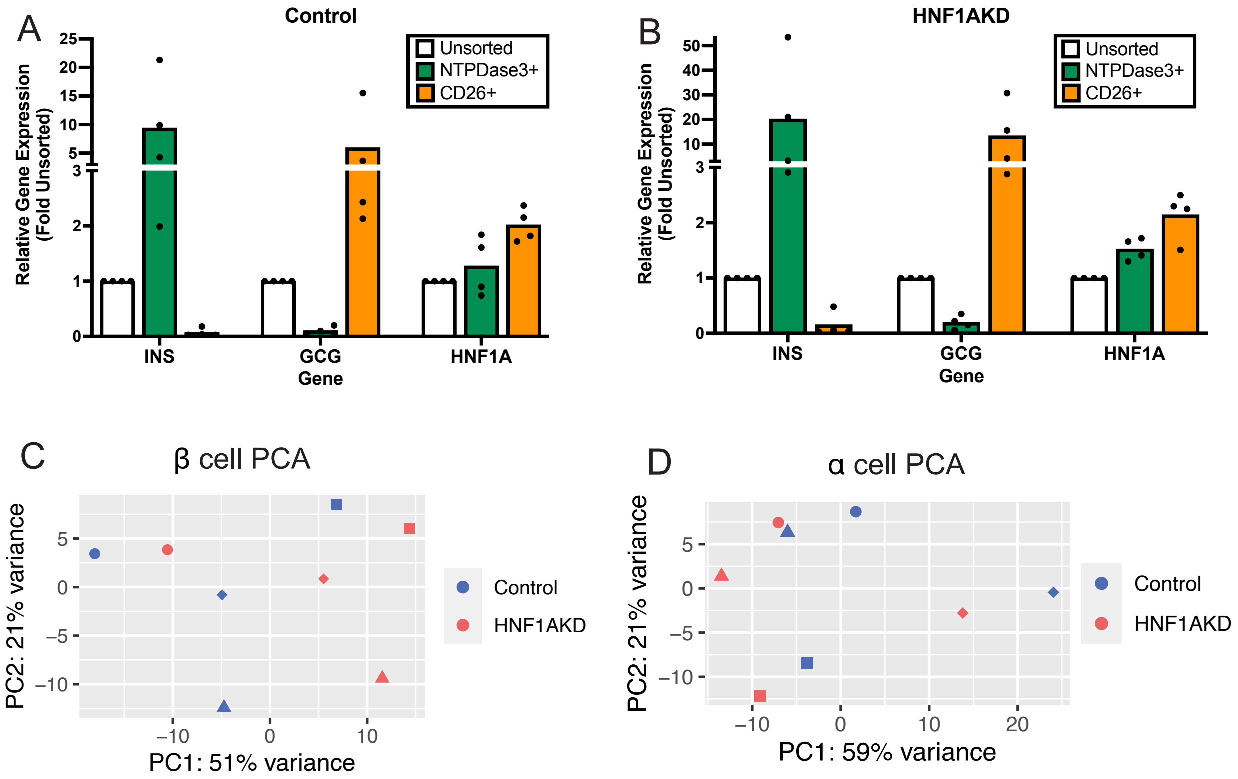

Figure S3. Fluorescence-activated cell sorting (FACS) of pseudoislets enriches β and α cell populations for RNA sequencing analysis. (A-B) qRT-PCR demonstrated enrichment of insulin (*INS*) with depletion of glucagon (*GCG*) in $GFP^{+}HPi2^{+}NTPDase3^{+}$ versus unsorted fractions and enrichment of glucagon with depletion of insulin in $GFP^{+}HPi2^{+}CD26^{+}$ fractions from sorted (A) Control and (B) HNF1AKD pseudoislet samples (n=4 donors). (C-D) Principal Component Analysis (PCA) of RNA sequencing results from sorted (C) β ($NTPDase3^{+}CD26^{-}$) and (D) α ($CD26^{+}NTPDase3^{-}$) cells of Control (blue) and HNF1AKD (salmon) pseudoislet samples (n=4 donors; distinct donors are indicated by different shapes).

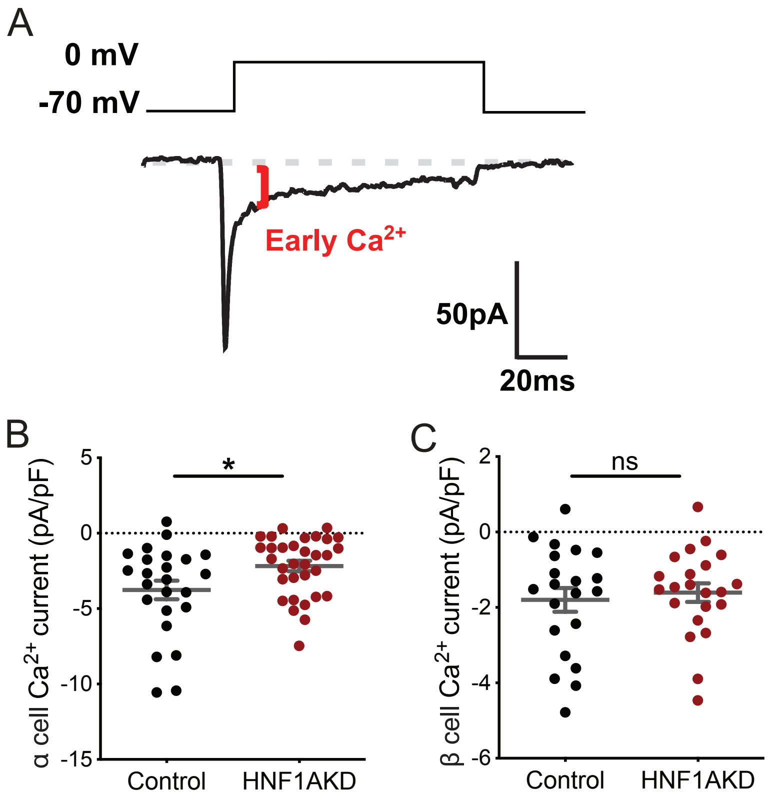

Figure S4. In vitro assessment of calcium flux in primary human pseudoislets 5 days after transduction with lenti-Control-shRNA ("Control") or lenti-HNF1A-shRNA ("HNF1AKD"). (A) Representative patch clamp electrophysiology trace of current recordings from islet cells. Early calcium currents from (B) α cells and (C) β cells (n=18-32 cells per condition from 3 human donors). Error bars represent SEM. Two-tailed t-tests were used to generate p-values; $^*P<0.05$, ns= not statistically significant.

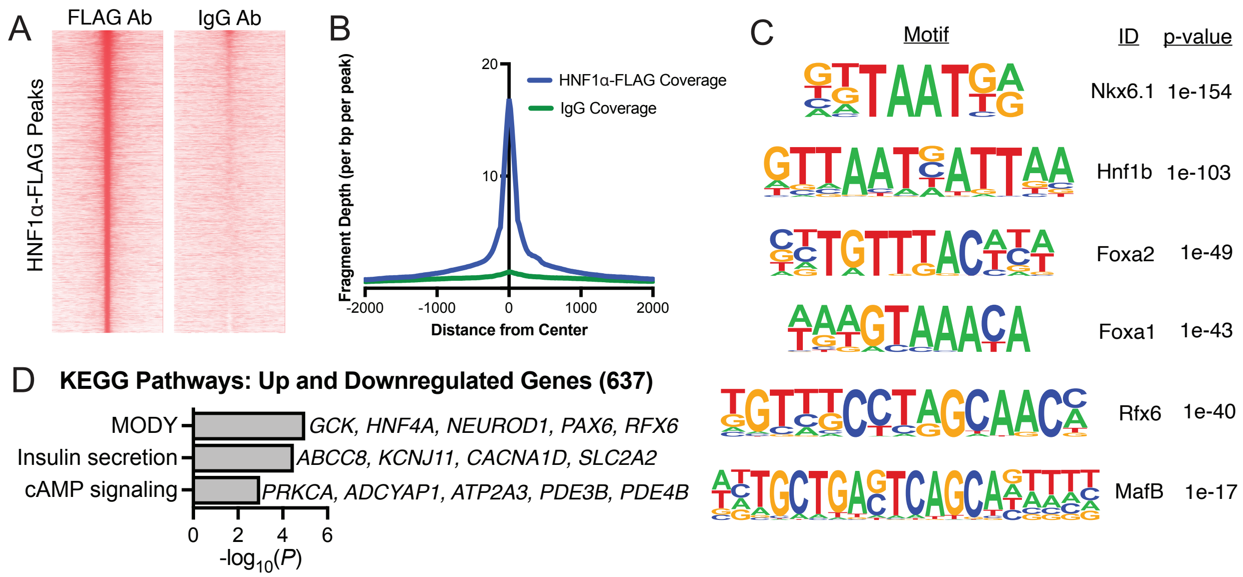

Figure S5. CUT&RUN identifies direct binding targets of HNF1α in primary human islet cells. (A) Heatmap visualization of CUT&RUN peaks and (B) histogram plots of averaged reads demonstrated enrichment of read densities in HNF1α-FLAG CUT&RUN library peak centers versus minimal enrichment at these sites for IgG control samples. (C) HOMER motif analysis identified that HNF1α-FLAG-bound genomic peaks were significantly enriched for pancreatic transcription factor motifs (NKX6.1, HNF1b, FOXA2, FOXA1, RFX6 and MAFB) previously shown to be enriched alongside HNF1α in pancreatic islet enhancer clusters. (D) KEGG pathways enriched in putative HNF1α target genes, which were identified by the intersection of HNF1α-FLAG CUT&RUN and HNF1AKD RNA-seq gene sets.

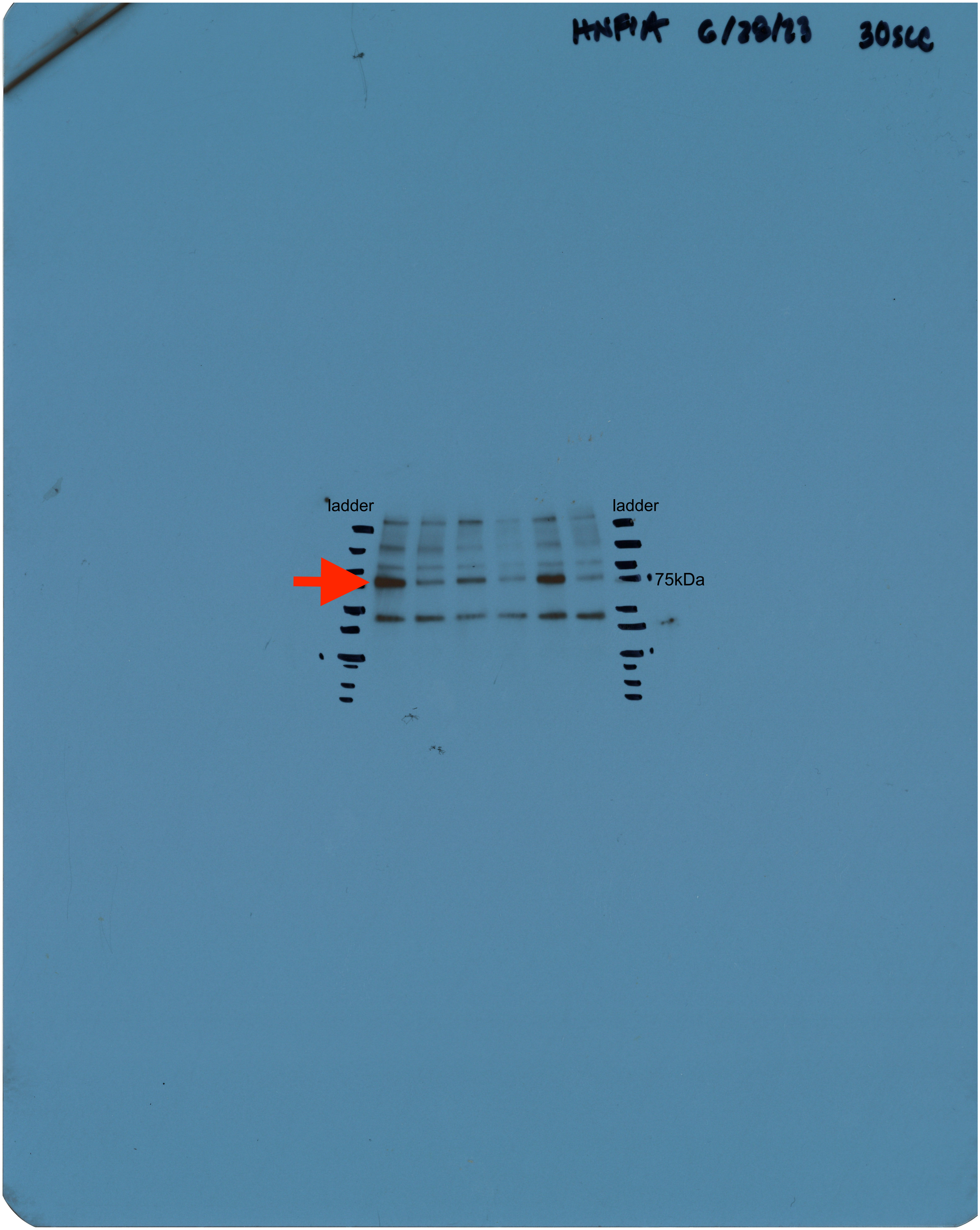
HNF1A 6/28/23 30sec
ladder
ladder
75kDa

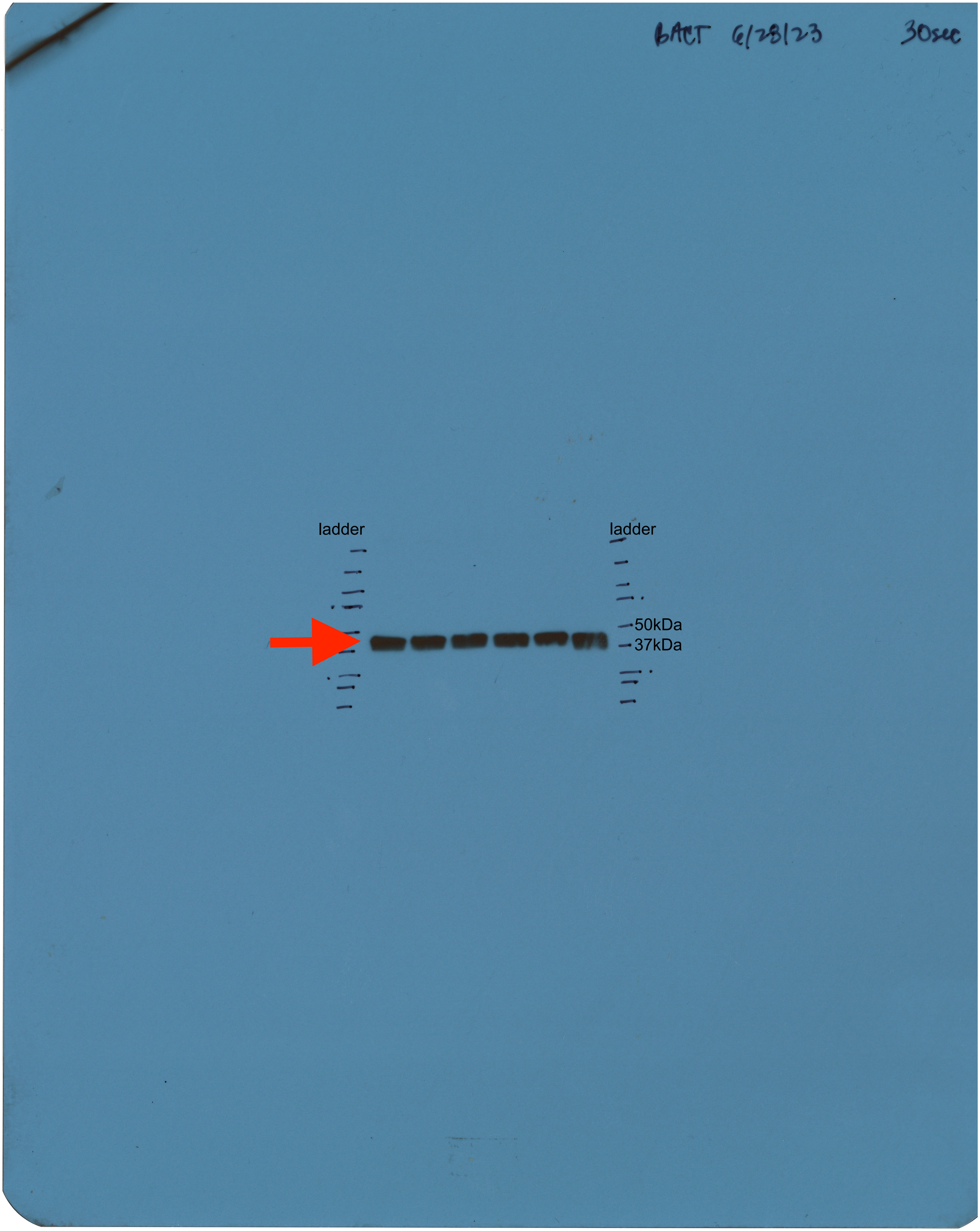
bACT 6/28/23
30sec
ladder
ladder
50kDa
37kDa
